# Egg excretion patterns of soil-transmitted helminth infections in humans following albendazole-ivermectin and albendazole treatment

**DOI:** 10.1371/journal.pntd.0012073

**Published:** 2024-03-22

**Authors:** Sophie Welsche, Pierre H. H. Schneeberger, Jan Hattendorf, Somphou Sayasone, Eveline Hürlimann, Jennifer Keiser

**Affiliations:** 1 Swiss Tropical and Public Health Institute, Allschwil, Switzerland; 2 University of Basel, Basel, Switzerland; 3 Lao Tropical and Public Health Institute, Vientiane, Lao PDR; University of Passo Fundo: Universidade de Passo Fundo, BRAZIL

## Abstract

**Background:**

Control efforts of soil-transmitted helminthiases rely primarily on large scale administration of anthelminthic drugs. The assessment of drug efficacies and understanding of drug behavior is pivotal to the evaluation of treatment successes, both in preventive chemo-therapy programs as well as in research of novel treatment options. The current WHO guidelines recommend an interval of 14–21 days between the treatment and follow-up, yet no in-depth analysis of egg excretion patterns of *Trichuris trichiura* after treatment has been conducted to date.

**Methods:**

Within the framework of a multi-country trial to assess the efficacy and safety of albendazole-ivermectin combination therapy vs albendazole monotherapy against *T*. *trichiura* infections, we conducted a study collecting daily stool samples over the period of 28 days post-treatment in 87 participants in Pak Khan, Lao PDR. Egg counts were derived by duplicate Kato-Katz on-site for *T*. *trichiura*, hookworm and *Ascaris lumbricoides* and stool sample aliquots were subsequently analyzed by qPCR for the detection of *T*. *trichiura* infections. Sensitivity and specificity was calculated for each day separately using data derived by Kato-Katz to determine the optimal timepoint at which to assess drug efficacy.

**Results:**

Egg excretion patterns varied across treatment arms. For *T*. *trichiura*, only the albendazole-ivermectin treatment led to a considerable reduction in mean egg counts, whereas both treatments reduced hookworm egg counts and *A*. *lumbricoides* were cleared in all participants after day 7. For *T*. *trichiura*, we found sensitivity to be highest at days 18 and 22 when using egg counts as outcome and days 19 and 24 when using qPCR. Specificity was high (>0.9) from day 14 onwards. For hookworm, the highest sensitivity and specificity were found at days 17 and 25, respectively.

**Conclusions:**

Based on our study, the ideal time period to assess drug efficacy for soil-transmitted helminth infections would be between day 18 and 24. The current WHO recommendation of 14 to 21 days is likely to yield acceptable outcome measures for soil-transmitted helminth infections.

**Trial registration:**

NCT03527732.

## Author summary

Whipworm (*T*. *trichiura*), hookworm and roundworm (*A*. *lumbricoides*) infections are highly common worldwide. To reduce the morbidity caused by these infections, hundreds of million deworming drugs are given to children every year. To assess how good deworming drugs work it is recommended to microscopically analyze stool samples 14–21 days after treatment, however the evidence base for this recommendation is scarce. In Lao PDR we conducted a study collecting daily stool samples from 87 participants for 28 days post-treatment with two deworming drugs, single albendazole and co-administered albendazole-ivermectin. We counted the eggs of *T*. *trichiura*, hookworm and *A*. *lumbricoides* of each sample by microscopy and calculated the daily proportions of correctly classified negative or positive individuals according to considered real negatives and positives. We used these measures to find the optimal timepoint at which drug efficacy should be assessed. We found that only albendazole-ivermectin reduced the egg counts of whipworm infection whereas both treatments reduced hookworm egg counts and *A*. *lumbricoides* worms were absent in all participants after day 7. The best time points to evaluate the activity of the deworming drugs with microscopy were found at days 18 and 22 for whipworm, and at days 17 and 25 for hookworm, respectively.

## Introduction

Infections with soil-transmitted helminths (STHs) take up a major part of the disease burden caused by all neglected tropical diseases (NTDs). The three major causative agents *Trichuris trichiura*, *Ascaris lumbricoides* and the hookworms are often highly pervasive in their endemic regions and give rise to comparatively inconspicuous yet debilitating effects in infected individuals [1,2]. Control programs based on preventive chemotherapy with albendazole or mebendazole have been established for decades, with a global control strategy being widely promoted since the 1990s [3]. Yet still 1.5 billion people remain infected to date [4]. In their targets for STH control programs, the World Health Organization (WHO) has set a goal for 2030 to achieve and maintain elimination of morbidity due to STH infection in pre-school-aged children (pre-SAC) and SAC by reducing prevalence of moderate to heavy infection intensities in this age group to <2% in endemic countries [5]. The two benzimidazoles are the standard drugs distributed in preventive chemotherapy schemes. Albendazole and mebendazole are good candidates for mass drug administration (MDA) programs due to their cost-effectiveness, easy formulation and excellent safety profile. Both drugs are efficacious against *A*. *lumbricoides* and albendazole has an acceptable efficacy against hookworm, yet neither drug has acceptable treatment outcomes against *T*. *trichiura* [6,7]. These drugs are and have been an important tool for the reduction of morbidity by preventive chemotherapy [8], yet for moving towards sustainably interrupting transmission, improved treatment options are desperately needed [9,10]. Recently, increasing evidence has been found that co-administering ivermectin with albendazole yields superior treatment efficacies compared to albendazole monotherapy against *T*. *trichiura*, and the WHO has added the combination to the Essential Medicines List in 2017 [11,12].

Kato-Katz microscopy is the current standard method to determine STH infection status by counting helminth eggs shed in stool [13]. This method is both fast and can be done with comparatively simple and easily attainable equipment, making it ideal for the widespread use in low-and middle-income countries and remote settings. However, the quality and accuracy of results derived by microscopy is highly dependent on the experience and skills of the laboratory technicians performing the diagnostics and may vary among different laboratories [14,15]. Furthermore, low infection intensities may often be missed and thus poor sensitivity has been consistently reported [16–18]. An alternative approach is the diagnosis by real-time quantitative polymerase chain reaction (qPCR), which has been shown to be more sensitive [19–21]. Nevertheless, qPCR has not replaced Kato-Katz as the gold standard diagnostic method mainly due to its costliness and requirement for both sophisticated equipment as well as highly trained staff [22]. To date, no standardized protocol has been established, which may lead to great inter - laboratory variation due to different extraction methods, reagents and even copy number thresholds to determine a positive result.

To assess the efficacy of anthelminthic drugs, generalizable standard guidelines are a prerequisite to ensure the comparability and robustness of generated data across sites and countries. Drug efficacy assessment is important both for the investigation of novel drugs as well as for the evaluation of treatment successes in control programs. A crucial aspect is the post-treatment time point at which to determine the efficacy [23]. A consensus has been established and the WHO accordingly recommends assessment at 14–21 days post-treatment [13]. However, the time at which efficacy is assessed varies across studies [14,24]. The post-treatment assessment time point should both leave enough time for remaining eggs to be cleared from the body yet early enough to cut down on study time and to avoid under-estimation of drug efficacy due to potential re-infection. Only few studies have investigated egg excretion patterns for helminths to date, none of which focused on *T*. *trichiura*, as no efficacious treatment has been available until now [23,25,26]. To close this knowledge gap, we collected daily stool samples from STH-positive individuals over 28 days after they received anthelminthic treatment. This sub-study was conducted within the framework of a multi-country study assessing the efficacy of the drug combination albendazole-ivermectin in comparison to the standard treatment of albendazole monotherapy against *T*. *trichiura* infections. The randomized controlled trial was set in Tanzania, Côte d’Ivoire and Lao PDR and showed superiority of the combination therapy in two settings (Tanzania and Lao PDR) [12]. By closely following a sub-population of the study in Lao PDR after treatment, we aimed at describing the egg excretion patterns of the three major STH species with a focus on *T*. *trichiura* infections in order to shed light on the question which time window is ideal for the assessment of the currently best treatment options. Furthermore, to our knowledge, qPCR has not been used to analyze daily samples collected over a period of roughly one month. Our data may provide further insights on the feasibility of utilizing qPCR for the diagnosis of *T*. *trichiura* infections. Lastly, our study provides a better understanding on the pharmacodynamics of the recently introduced combination albendazole-ivermectin in the treatment of STH infections.

## Materials and methods

### Ethics statement

The trial received ethical clearance from the ethical committee of Northwest and Central Switzerland (EKNZ, BASEC Nr. Req-2018-00494) and the ethical committees of the trial countries (for Lao PDR: 093/NECHR). All participants provided written informed consent. For minors under the age of 18 years, a parent or guardian signed the consent form and the underage participants provided additional oral consent. The trial was registered on ClinicalTrials.gov (NCT03527732).

### Study area and population

The sub-study on daily sampling was conducted within the framework of a multi-country randomized controlled trial to investigate the efficacy and safety of co-administered albendazole-ivermectin vs. albendazole monotherapy against infections with *T*. *trichiura*. The study was conducted between September 2018 and June 2020 in three settings: Pemba Island, Tanzania in East Africa, Côte d’Ivoire in West Africa and Lao PDR in Southeast Asia. Details on study rationale, procedures and results are described in previously published articles [12,27,28]. The trial was conducted in Lao PDR in 10 villages of Nambak district, Luang Prabang province in Northern Lao PDR. The daily sampling sub-study took place in the village of Pak Khan due to an adequate number of participating inhabitants and good compliance during the screening phase. In Pak Khan, 88 participants aged 6–60 years were enrolled in the study with 87 having provided samples post-treatment for the daily sampling sub-study. In view of the increased logistical requirements and high demands to compliance of the participants due to repeated sampling over a longer period, and difficult accessibility of the study villages, the sub-study was conducted in Pak Khan village only. Consequently, the sample size was determined by the number of inhabitants, the local baseline infection rate for *T*. *trichiura* and compliance rate of Pak Khan village, but still exceeded the targeted minimum of 50 participants as in other similar studies [23].

### Trial procedures and sampling

At baseline, participants were asked to provide a total of two morning stool samples within a period of five days, which were used to determine the infection status. Participants meeting the inclusion criteria were invited for randomization and treatment. A threshold of 100 *T*. *trichiura* eggs per gram (epg) was set as qualification for inclusion; information on all inclusion criteria are defined elsewhere [12]. Prior to the administration of the study treatment (i.e. either albendazole-ivermectin or albendazole monotherapy), an information session was held in Pak Khan both with village authorities and study participants to explain both the clinical trial as well as the daily sampling sub-study. After the administration of the treatment, the 87 study participants living in Pak Khan were visited by the study team daily for a period of 28 days for the collection of stool samples. The span of 28 days for the sampling period was selected to allow sufficient time to observe potential recurrence of egg excretion without the risk for confounding by re-infection as all three assessed STH species require over a month from infection to oviposition [1,29,30]. Daily sampling took place between 5^th^ May and 1^st^ June 2019. At the end of each of the four weeks, participants having provided a stool sample received a small gift in the form of cooking oil. After completion of the 28 days, participants continued to be followed within the scope of the multi-country trial according to the protocol [27].

### Laboratory procedures

#### Kato-Katz microscopy and aliquotting

The stool samples collected in Pak Khan were transported to the field laboratory set at the Nambak hospital. From each sample, duplicate Kato-Katz slides were prepared and read by skilled laboratory technicians. A detailed description of the Kato-Katz diagnostics is noted elsewhere [12]. Egg counts for the STH species *T*. *trichiura*, *A*. *lumbricoides* and hookworm were recorded. The baseline epg for each individual was calculated as the arithmetic mean from both stool samples (i.e. quadruplicate Kato-Katz slides) x 24. For the daily sampling, the epg for each individual was calculated as the arithmetic mean from the duplicate Kato-Katz slides made from each of the samples x 24. From every sample, a small amount of feces was transferred into a 2ml screw cap cryotube using a UV sterilized plastic spatula. The aliquots were immediately frozen at -20°C. At the end of the trial, the aliquots were shipped to Swiss TPH in Basel on dry ice. Upon arrival, the tubes were immediately transferred to -20°C freezers for storage until they were used for DNA extraction and subsequent qPCR.

#### DNA extraction and qPCR

The STH DNA was extracted from the frozen stool aliquots using a QiAamp DNA Kit (Qiagen; Hilden, Germany) following a protocol using a garnet bead-beating approach developed by Kaisar et al. [31]. Minor modifications to the protocol were applied as validated by our group for previous projects [32] and frozen stool was used instead of stool fixated in ethanol and stored at room temperature. After extraction, real-time qPCR was conducted on a CFX Maestro system (Bio-Rad Laboratories, Hercules, CA, USA) using Taqman Gene Expression Master Mix (Thermo Fisher Scientific, Waltham, MA, USA) and target primers and probes for *T*. *trichiura* (Eurofins Genomics, Ebersberg, Germany). 96-well qPCR plate plans were set for the optimal usage of well space with samples of the same day grouped on one plate and 50 negative controls randomly distributed across plates. Ten positive controls with STH sequence insert containing plasmids were included on each plate. Standard curves were generated using a serial dilution of a plasmid containing the target sequence at known concentrations. For the PCR reactions, DNA was diluted 10-fold and each 10 μl reaction consisted of 0.3 μM of forward and reverse primer each and 0.1 μM probe for *T*. *trichiura* respectively. In addition, 5 μl of master mix, 0.9 μl of ultrapure water and 2 μl of DNA template were added. Cycling conditions were 2 min at 50°C, 10 min at 95°C followed by 40 cycles at 95°C for 15 sec and 60°C for 1 min. The fluorescence detection threshold was set at 1500 RFU. Reactions were conducted in duplicate, primers and probes are summarized in S1 Table.

### Statistical analysis

Kato-Katz data were double entered into a database using EpiInfo (v3.5.4) and checked for consistency using Beyond Compare 4 (Scooter Software Inc., Madison, Wisconsin). Data of the qPCR amplification were standardized using the standard curves of CFX Maestro Software. All data were analyzed using Stata 16 (StataCorp LLC), Microsoft Excel and R (v4.0.3). The egg excretion patterns derived by Kato-Katz for *T*. *trichiura*, *A*. *lumbricoides* and hookworm were calculated as mean egg counts of quadruplicate Kato-Katz slides for baseline samples and duplicate slides for post-treatment samples and multiplied by a factor of 24 to obtain epg values. All qPCR assays not resulting in an amplification curve were considered negative and equaled to zero copy numbers. The patterns of copy numbers were calculated from the mean of duplicate qPCR assays.

The following formulas for geometric and arithmetic mean were used [33]:

Geometric mean was calculated as
$$GM\left(x_{1},\;\ldots ,\;x_{n}\right)=e^{(\frac{1}{n}\sum_{i=1}^{n}\text{log}(x_{i}+1))}-1.$$
Arithmetic mean was calculated as
$$AM\left(x_{1},\;\ldots ,\;x_{n}\right)=\frac{1}{n}\sum_{i=1}^{n}x_{i}.$$

Egg excretion patterns were further described by calculating daily geometric and arithmetic egg reduction rates (ERRs) by treatment arm for *T. trichiura* infection using the same formulas as in the efficacy study [12]. To determine the optimal time point at which to assess anthelminthic efficacy post-treatment, the sensitivity/specificity was calculated for each day. The starting point of the analysis was set at day 10, as egg excretion was expected to have stabilized then with no more adult worms and residual eggs getting expulsed [34]. Only participants having provided at least 7 samples between day 10 and day 28 were included in the analysis to avoid skewing results due to missing data. Infection status was considered to be true positive (TP) if between day 11 and day 28 i) two samples had an egg count of at least 48 epg, or ii) if at least three samples were found positive for eggs. Samples not defined as true positive were considered true negative (TN). Subsequently, the ratio of samples found positive to true positives was calculated per day resulting in a sensitivity curve, as well as the ratio of negatives to true negatives for the specificity curve, accordingly. The analysis was conducted based on Kato-Katz results for *T*. *trichiura* and hookworm only, as *A*. *lumbricoides* was completely cleared in both treatment arms during this time window.

## Results

The baseline characteristics of the 87 study subjects are summarized in Table 1. All participants were positive for *T*. *trichiura* with an egg count of ≥100 epg, given the inclusion criterion of the randomized controlled trial. Out of 87 participants, 6 harbored a single infection with *T*. *trichiura*, 50 were infected with two species (6 *T*. *trichiura* and *A*. *lumbricoides*, 44 *T*. *trichiura* and hookworm) and 31 participants were infected with all three species.

**Table 1:** Baseline characteristics of participants by treatment arm.

	Albendazole-ivermectin (N = 42)	Albendazole monotherapy (N = 45)
Age, years	29.1 (2.6)	26.2 (2.6)
Age group ‡		
School-aged children (6–12)	13 (31%)	16 (36%)
Adolescents and young adults (13–24)	3 (7%)	8 (18%)
Adults (25–60)	26 (62%)	21 (47%)
Sex assigned at birth		
Female	26 (62%)	21 (47%)
Male	16 (38%)	24 (53%)
*T*. *trichiura* infection		
Geometric mean epg	498.2	480.2
Arithmetic mean epg	992.4	753.1
Infection intensity †		
light	30 (71%)	34 (76%)
moderate	12 (29%)	11 (24%)
heavy	0	0
Hookworm infection		
Infected	38	37
Geometric mean epg	741.1	378.1
Arithmetic mean epg	1378.3	1475.4
Infection intensity *		
light	30 (79%)	30 (81%)
moderate	5 (13%)	4 (11%)
heavy	3 (8%)	3 (8%)
*A*. *lumbricoides* infection		
Infected	18	19
Geometric mean epg	2514.0	1509.1
Arithmetic mean epg	7134.0	6808.7
Infection intensity **		
light	11 (61%)	13 (68%)
moderate	7 (39%)	6 (32%)
heavy	0	0
Single *T*. *trichiura* infection	1	5
Polyparasitism: two species	26	24
Polyparasitism: three species	15	16

Data are mean (SD) or N (%) unless otherwise stated. Epg = eggs per gram of stool.

^‡^ Age groups were defined according to Medical Subject Heading (MeSH) categorization.

^†^ *T*. *trichiura* infection intensity was classified into light (1–999 epg), moderate (1000–9999 epg) and heavy (≥10000 epg).

* Hookworm infection intensity was classified into light (1–1999 epg), moderate (2000–3999 epg) and heavy (≥4000 epg).

** *A*. *lumbricoides* infection intensity was classified into light (1–4999 epg), moderate (5000–49999 epg) and heavy (≥50000 epg).

**Fig 1:**
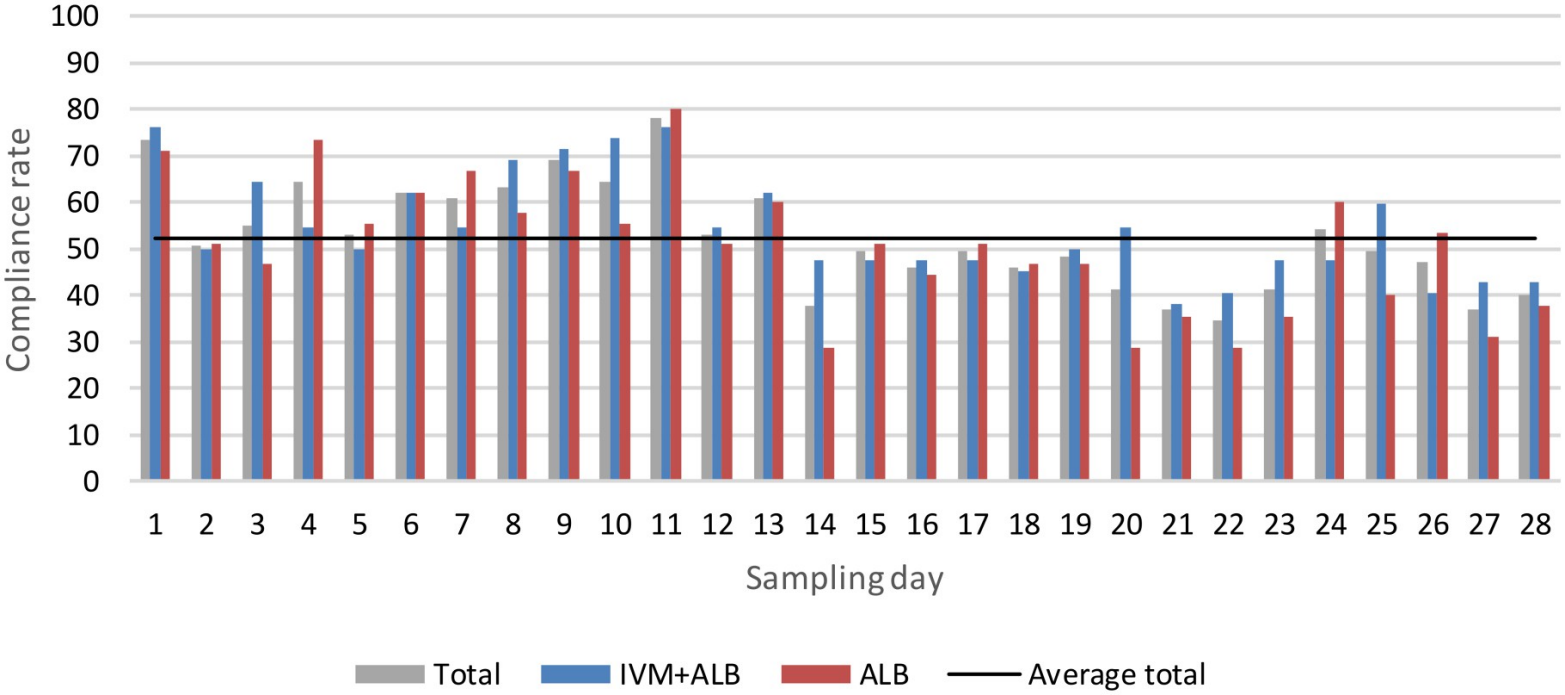
Compliance of study participants. Data is presented as fraction of participants having provided a stool sample each day. 100% = 87 participants, average total: 52.4 (SD 11.6); Average IVM+ALB: 54.3 (SD 11.2); Average ALB: 50.6 (SD 14.1).

In total, 1276 stool samples were collected over the period of 28 days, with an arithmetic mean of 15.7 (SD 6.9) samples per participant (and no difference across treatment arm: albendazole-ivermectin: 16.2 (SD 6.9); albendazole: 15.2 (SD 6.9)). Compliance remained relatively stable, 52.4% (SD 11.6) of participants provided a sample each day on average, yet the number of samples provided decreased slightly towards the end of the study. The daily compliance over the whole sampling period is depicted in Fig 1.

### Egg excretion patterns

#### *T*. *trichiura*

**Fig 2:**
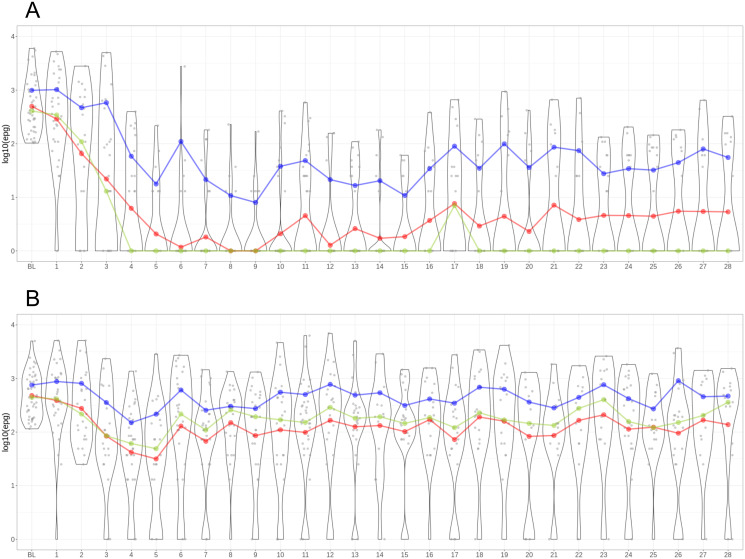
*T*. *trichiura* egg excretion pattern with egg counts derived from Kato-Katz microscopy from participants in the albendazole-ivermectin treatment (A) and albendazole (B) group at logarithmic scale. Points represent egg counts from a single sample, lines represent the daily mean eggs per gram (epg), violin plots show the distribution of epg per day. Red line: geometric mean. Blue line: arithmetic mean. Green line: median. BL = baseline, 1–28 = days post-treatment.

The distribution of egg counts per day over the 28 day sampling period are depicted in Fig 2A and 2B for the albendazole-ivermectin combination and the albendazole monotherapy treatment arms, respectively.

Among the participants having received the combination treatment of albendazole-ivermectin, the mean epg decreased noticeably within the first week after treatment, from 498 and 992 (log_10_ 2.7 and 3.0) at baseline and reaching its lowest point on day 8 and 9 for the geometric and arithmetic mean respectively, at a geometric mean of 0.8 (log_10_ 0) on day 8 and an arithmetic mean of 8 (log_10_ 0.90) on day 9 (Fig 2A). Both the geometric and arithmetic mean however increased again subsequently. During the last week of sampling (days 22–28), the arithmetic mean stayed stable between 28 to 80 epg (log_10_ 1.4 to 1.9) and the geometric mean at 4 to 6 epg (log_10_ 0.6 to 0.7). The arithmetic mean egg reduction rate (ERR) ranged between 91.9% and 97.2% and the geometric mean egg ERR ranged between 99.1% and 98.9% during days 22–28.

At baseline, the geometric mean egg count of the albendazole-treated participants was 480 epg and the arithmetic mean 753 epg (log_10_ 2.7 and 2.9). The mean epg of albendazole-treated participants dropped slightly during the 28 day sampling period, with the lowest values observed on day 4 at an arithmetic mean of 150 (log_10_ 2.2) and day 5 for a geometric mean of 31.5 (log_10_ 1.5) (Fig 2B). By the last week of the sampling (days 22–28), the arithmetic mean egg counts were similar to the baseline values: 271 to 909 epg (log_10_ 2.4 to 3.0) compared to 753 (log_10_ 2.9) at baseline. The geometric mean at 95 to 210 epg (log_10_ 2.0 to 2.3) was slightly lower than the baseline value of 480 epg (log_10_ 2.7). Correspondingly, the arithmetic mean ERR ranged between negative values and 64.1%, whereas the geometric mean ERRs ranged between 56.2% and 80.2%.

When considering the development of egg excretion at the individual level, the results showed that some individuals, especially among the combination therapy treatment arm, consistently stayed egg-negative with a maximum of one outlier after the daily egg counts had dropped to 0 on several consecutive days. This was the case for 22 participants in the albendazole-ivermectin group, and for 2 participants in the albendazole monotherapy group. In contrast, other participants’ egg counts showed a resurgence after an initial drop, in some cases to 0 over several days. Finally, for some participants egg counts stayed around the same level as before treatment throughout the observation period, which almost exclusively occurred among the albendazole monotherapy arm.

#### Hookworm

Both treatment regimens resulted in a clear reduction of mean egg counts. At baseline, the geometric mean egg count was 741 epg and the arithmetic mean 1378 epg among the participants in the combination treatment arm (log_10_ 2.9 and 3,1), and participants in the albendazole monotherapy treatment arm had a geometric mean of 378 epg and an arithmetic mean of 1475 epg (log_10_ 2.6 and 3.2). The lowest means were reached on days 4 and 5, with a geometric mean of 1.2 and 1.5 epg (log_10_ 0.1 and 0.2) and an arithmetic mean of 23.4 and 12.6 epg (log_10_ 1.4 and 1.1), on days 4 and 5, respectively, for the combination therapy arm. The results for the monotherapy arm on days 4 and 5 were at a geometric mean of 0.0 and 0.3 (log_10_ 0 and 0) and an arithmetic mean of 0.0 and 9.8 epg (log_10_ 0.0 and 1.0), respectively. Following the initial drop, the mean egg counts increased again across both treatment groups. In the albendazole monotherapy treatment arm, the geometric mean is consistently lower throughout the sampling period compared to the combination treatment arm, which is also reflected in the median as well as in the distribution of egg counts (S1 Fig).

#### *A*. *lumbricoides*

A sustained and complete reduction of egg counts was observed in all study individuals infected with *A*. *lumbricoides* across both treatment arms (S2 Fig). At baseline, participants in the combination treatment arm had geometric mean egg counts of 2514 epg and an arithmetic mean of 7134 epg (log_10_ 3.4 and 3.9), participants in the monotherapy treatment arm had a geometric mean of 1509 epg and an arithmetic mean of 6809 epg (log_10_ 3.2 and 3.8). Geometric and arithmetic mean as well as median dropped to 0 epg on day 5 for the albendazole-ivermectin combination treatment and remained there for the remaining sampling period with one outlier on day 10. Among the individuals having received the albendazole monotherapy, the same drop to 0 epg can be observed slightly later on day 8.

### Optimal timepoint for assessment of anthelminthic efficacy

#### *T*. *trichiura*

Daily egg counts derived by Kato-Katz were considered from 58 participants, who had provided at least 7 samples between day 10 and 28. 40 participants fell into the true positive category (at least 2 samples with more than 48 epg or 3 positive samples between day 11 to 28), and 18 were considered true negative.

**Fig 3:**
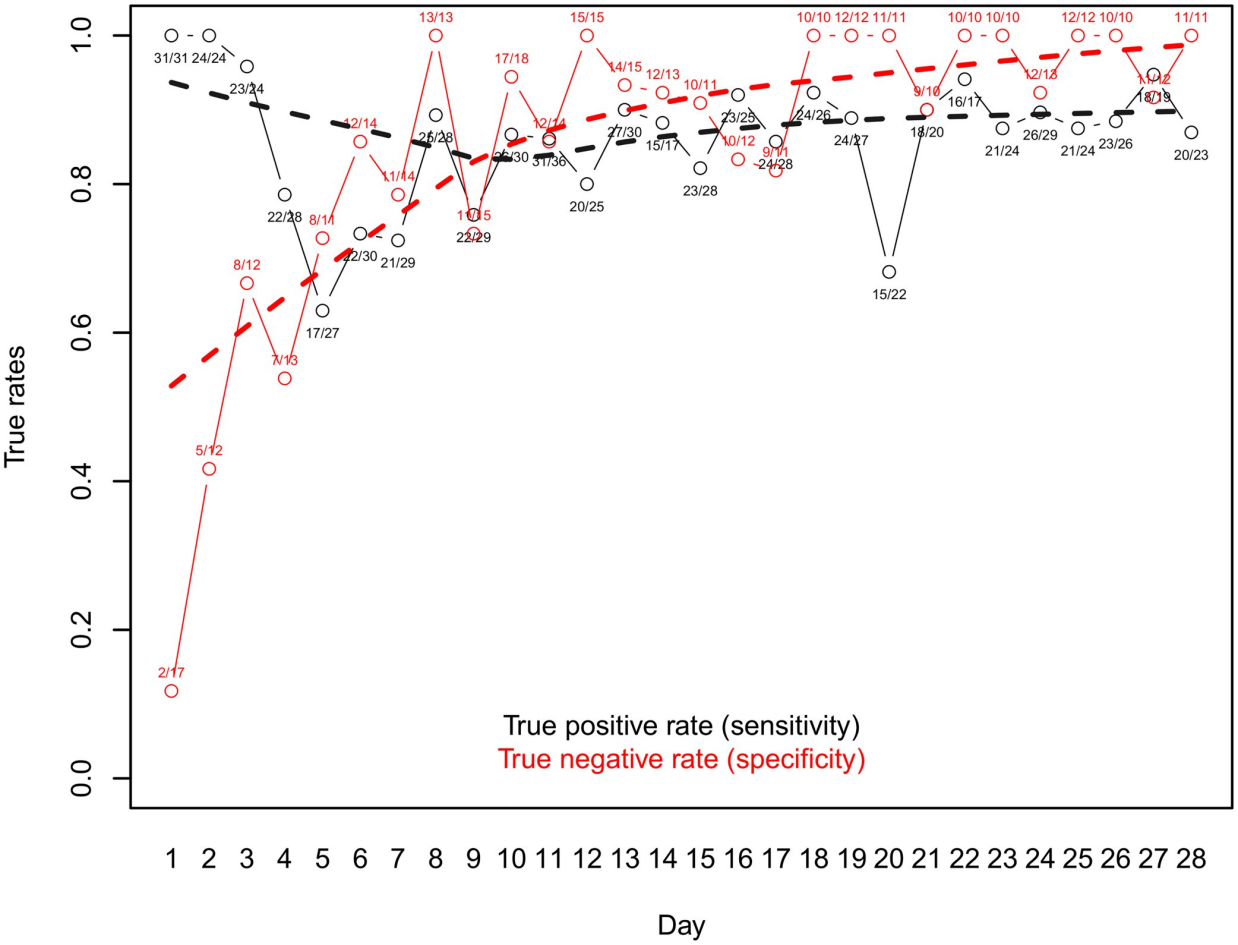
Sensitivity and specificity analysis per day post-treatment for *T*. *trichiura* results derived from duplicate Kato-Katz slides. Rates are calculated from the ratio of number of positive results vs number of true positives, as defined per the analysis.

The sensitivity and specificity curves of the resulting sub-group is depicted in Fig 3, with daily sensitivity being calculated as the ratio of apparent positive to defined true positive results found on a given day, and daily specificity calculated accordingly as the ratio of apparent negative to defined true negative results, with true negative defined as not classified true positive. The best sensitivity was observed on days 18 and 22.

#### Hookworm

Out of the 75 participants positive for hookworm at baseline, 50 provided at least 7 samples between day 10 and 28. Out of those, 23 participants were classified as true positive (at least 2 samples with over 48 epg or 3 positive samples between days 11 and 28), and 27 were considered true negative. S3 Fig shows the resulting sensitivity and specificity curves. The highest sensitivity and specificity was observed at days 17 and 25.

### Analysis of stool samples for *T*. *trichiura* infection by qPCR

**Fig 4:**
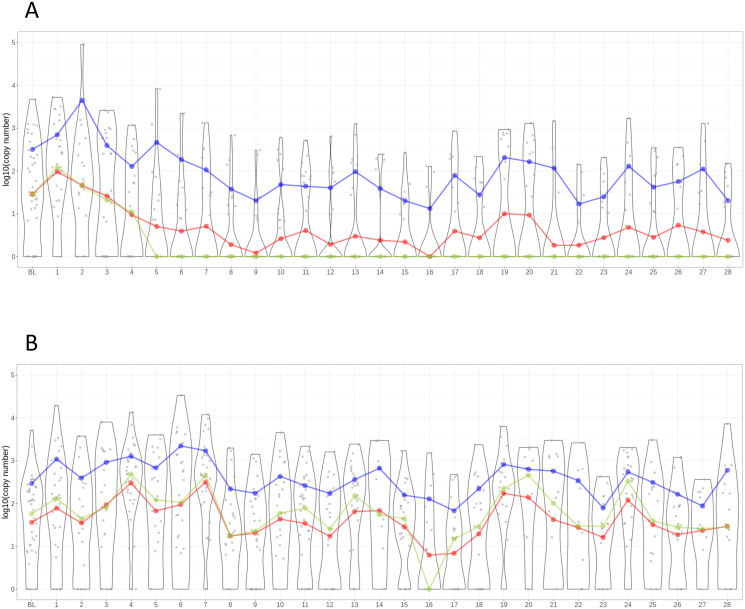
Daily pattern of *T*. *trichiura* copy numbers derived by qPCR from participants in the albendazole-ivermectin (A) albendazole monotherapy (B) treatment group at logarithmic scale. Points represent copy numbers from a single sample, lines represent the daily mean copy numbers, violin plots show the distribution of copy numbers per day. Red line: geometric mean. Blue line: arithmetic mean. Green line: median. BL = baseline, 1–28 = days post-treatment.

Aliquots of all stool samples used for the analysis with Kato-Katz on-site were subsequently assessed for *T*. *trichiura* infection by qPCR. The set of samples was complete with the exception of two baseline aliquots that were missing and could thus not be analyzed by qPCR. The resulting patterns of copy numbers per day are depicted in Fig 4A and 4B for participants in the albendazole-ivermectin combination treatment arm and the albendazole monotherapy arm, respectively.

The qPCR results mirror the overall trends of the Kato-Katz egg counts, most noticeably the difference between arm A and B and the decreased daily means and medians post-treatment among the samples from participants of arm A. However, the baseline results are less homogeneous, which also results in the baseline daily mean and median among arm A samples being lower than at later post-treatment timepoints, most noticeably at day one and two. Furthermore, the overall daily variation of copy numbers is higher than for the Kato-Katz egg counts.

For the albendazole-ivermectin group, the copy numbers at baseline amount to 29.1 and 317.4 (log_10_ 1.5 and 2.5) for geometric and arithmetic mean, respectively. The lowest values are observed on day 9, with 1.2 and 20.2 copy numbers (log_10_ 0.1 and 1.4) for geometric and arithmetic mean and on day 16, with 0.9 and 13.2 copy numbers (log_10_ 0 and 1.1) for geometric and arithmetic mean. Among the participants having received albendazole only, the geometric and arithmetic mean of the baseline results were 37.4 and 293.3 copy numbers (log_10_ 1.6 and 2.5). Unlike the Kato Katz results, there is no visible drop in copy numbers over the first few days post-treatment and the daily mean results stay around the same levels throughout the observation period. The lowest values were found on day 16 and 17, with a geometric mean of 6.2 and 6.9 (log_10_ 0.8 and 0.8) and an arithmetic mean of 127.8 and 67.9 copy numbers (log_10_ 2.1 and 1.8), respectively.

**Fig 5:**
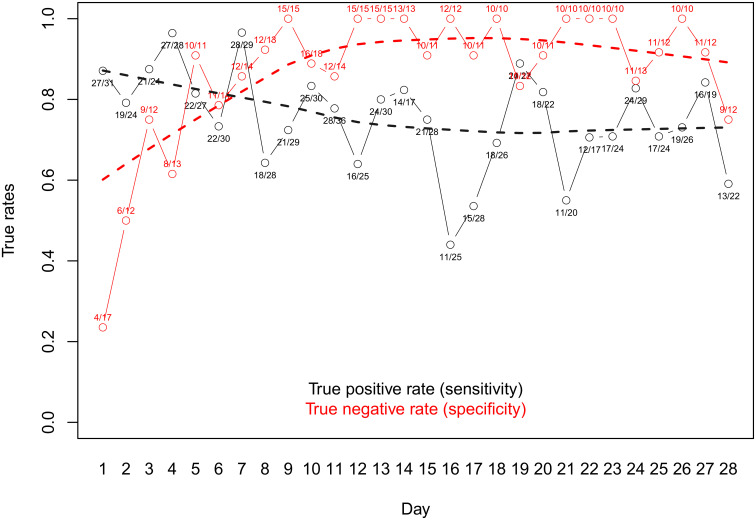
Sensitivity and specificity analysis per day post-treatment for *T. trichiura* results based on qPCR values. Ground truth was defined using egg counts derived from duplicate Kato-Katz slides. Rates were calculated from the ratio of the number of samples positive by qPCR over the number of true positives.

Using egg counts to define true positive and true negative samples as described above, we also assessed sensitivity and specificity using qPCR-based endpoint measure to quantify *T*. *trichiura* copy numbers. The sensitivity and specificity curves are shown in Fig 5. While specificity was high from day 14 onwards (> 0.9), sensitivity was highest on days 19 and 24, and was therefore correlating well with epg-based metrics. It is worthwhile mentioning that we found days with outlying sensitivity values (e.g. day 16 with a sensitivity value of 0.44) which is likely due to the methodological approach consisting of defining TP and TN values based on epg counts.

## Discussion

The treatment response observed within the population of Pak Khan to the two different treatment options matched the results of the overarching clinical trial with a significant higher efficacy observed for albendazole-ivermectin [12]. For *T*. *trichiura* infections, the low ERR and cure rate (CR) observed by Hürlimann et al. among the albendazole monotherapy arm are clearly mirrored in the egg excretion patterns [12]: neither mean (arithmetic and geometric) nor median egg counts dropped considerably. Furthermore, the egg counts of the majority of participants in the monotherapy arm did not drop to zero epg at any time during sampling. The daily egg counts derived from this sub-study demonstrates notably that a single dose of albendazole is not only unsuitable to cure participants but has only a small impact on infection intensity. The daily sampling approach sheds further light on the efficacy and pharmacodynamics of the albendazole-ivermectin combination treatment on infected individuals. Some individuals excreted no more eggs after the residual eggs were shed post-treatment, while others experienced a recurrence of egg shedding after an initial drop. The reason for the variable treatment efficacy of albendazole-ivermectin on *T*. *trichiura* is not known. A recent publication by Schneeberger et al. working with data from the same study population found a potential link of the gut microbiome to treatment outcome [35]. Albendazole and ivermectin have different modes of action, with benzimidazoles hampering cell functionality by inhibiting polymerization of tubulin in the cytoskeleton, whereas macrocyclic lactones lead to pharyngeal paralysis [36]. Accordingly, another explanation for the recurrence of egg shedding over time may be that some worms could have survived the treatment–both the monotherapy and the combination therapy- but were still affected, leading to an interruption of egg shedding during the first days after treatment. A recent study on the efficacy of triple dose albendazole for STH found recurrence of *T*. *trichiura* infections in initially cured participants at 6 weeks post-treatment [37]. The authors hypothesized that one explanation might be the survival of immature pre-adult worms or that worms may escape eradication from the body by embedding into the mucosa, which may allow them to recover initial paralysis [37].

For the two concomitant STH parasites, the egg excretion patterns were also in accordance with previous findings of treatment outcomes with the applied therapies [7,12,38]. Both treatment options led to a reduction in egg counts for individuals infected with hookworm. Nonetheless, several individuals in either arms still shed eggs throughout the sampling period, which indicate that both treatments are insufficient to cure hookworm infections. Albendazole is widely established as an excellent treatment against *A*. *lumbricoides*, which was clearly observable for infected study participants as well [6,7,26]. In both treatment arms, egg excretion dropped to zero at days 5–8 with no recurrence throughout the sampling period, which confirms the high ERRs and CRs consistently reported [10].

The time point of assessing treatment efficacy is highly critical to the representation of the true treatment outcome in measures of ERRs and CRs, for control programs as well as in research examining novel and improved treatment options. For *T*. *trichiura*, our analysis revealed that optimal sensitivity was reached at days 18 and 22, and days 19 and 24 when using egg counts and qPCR values, respectively, thus indicating a good agreement between the two detection methods. Specificity was high from day 14 onwards in both cases. For hookworms, optimal sensitivity and specificity was achieved on days 17 and 25 based on a single detection technique (egg counts). Even though there were considerable variations from day to day with occasional outliers, the smoothing lines allow for a more general interpretation. For *T*. *trichiura*, the true rates are fairly stable at a high level after day 11 and remain so until the end of the assessment at day 28. For hookworm, specificity remains consistently high after day 4, yet sensitivity steadily increases over time and TP rates stay generally more volatile and lower compared to *T*. *trichiura*. However, the sensitivity reaches 80% on day 10 and the incline of the trend becomes less steep thereafter. In a study assessing the egg output of helminths post-treatment with albendazole at several time points over the course of 44 days, Scherrer et al. (2009) did not find conclusive results on hookworm shedding. They stated that an assessment of efficacy against hookworm infections after the administration of albendazole at 2 to 3 weeks post-treatment seemed reasonable [23]. *A*. *lumbricoides* co-infections were less prevalent in the study population and the results showed no more egg excretion after day 4 (with one outlier on day 10) and day 7 in participants treated with albendazole-ivermectin combination therapy and albendazole monotherapy, respectively. Levecke et al. (2017) conducted an analysis of *A*. *lumbricoides* egg excretion after treatment with albendazole, recommending to wait until at least day 14 to assess drug efficacy [26]. Given the complete lack of excreted eggs later than 7 days post-treatment for albendazole monotherapy, our findings do not support the statement of the authors that the reduced efficacy found by Krücken et al. (2017) might be due to a biased ERR estimate rather than the potential emergence of anthelminthic resistance [39]. Of note, our sample size is comparatively small due to the lower prevalence of *A*. *lumbricoides* co-infections and the results should therefore be considered with care.

Our study provides the first in-depth assessment of the treatment outcome after administration of the currently best available treatment options on *T*. *trichiura* infections, namely albendazole-ivermectin. We identified day 18–24 as the optimal time window to evaluate treatment outcomes with albendazole and albendazole-ivermectin against soil-transmitted helminth infections. Putting our results in perspective with previous findings, we conclude that the currently recommended timeframe of assessing anthelminthic efficacy at 14 to 21 days post-treatment is likely to yield adequate CRs and ERRs for the combination chemotherapy for STH infections. However, our findings could be taken into consideration when revising the current recommendations with slightly shifting the follow up evaluation to a later time point.

A limitation of the study was the varying amount of samples collected per individual across the sampling period and the resulting missing points in the dataset. However, pooling the results of all samples per day and with the comparatively good and stable compliance, the available data is likely to provide an adequate representation of the actual egg excretion patterns. For the sensitivity analysis, participants with most missing data were not considered with the aim of further removing bias due to missing data. It is also worth highlighting that the egg excretion pattern is shaped by the treatment efficacy, which in turn is influenced by the administered drugs as well as the study participants’ infection intensity. For our study we have used the currently most efficacious treatment available albendazole-ivermectin and the commonly used monotherapy albendazole. The inclusion criteria of the overarching multi-country trial ensured that no ultra-light infections (<100 EPG) were included for *T*. *trichiura*. The majority of infections across all STH pathogens were light, whereas moderate intensities were fewer and heavy infections only occurred in some individuals infected with hookworm. In settings with higher or much lower infection intensities, the egg excretion patterns would change accordingly, and it is worth mentioning that a repetition in one of the African study settings of the clinical trial would have provided further insight. A different egg excretion pattern will also be observed using other treatments, as the pharmacodynamics of each treatment differs.

In conclusion, we analyzed for the first time the egg excretion patterns of *T*. *trichiura* and concomitant STH infections following two treatments - albendazole and a novel combination therapy, namely albendazole-ivermectin. The analysis of multiple samples did not only allow to compare the pharmacodynamics of both treatments but also to identify specific treatment phenotypes. Further studies are necessary to obtain a better understanding of why the treatment efficacy of albendazole-ivermectin against *T*. *trichiura* is variable. Our results confirm once more that the albendazole-ivermectin combination treatment has the potential to become a powerful tool for control programs based on preventive chemotherapy. The evidence of this study supports the assumption that assessing anthelminthic treatment outcomes at the currently suggested time window can yield to an accurate representation of the true efficacy, yet our findings should be taken into consideration when revising the current guideline.

## Supporting information

S1 Table Primers and probes used for the identification of *Trichuris trichiura* via real-time quantitative PCR. (DOCX)

S2 Table Underlying raw data. (CSV)

S1 Fig Hookworm egg excretion pattern with egg counts derived from Kato-Katz microscopy from participants in the albendazole-ivermectin (A) and albendazole (B) treatment groups at logarithmic scale. Points represent egg counts from a single sample, lines represent the daily mean eggs per gram (EPG), violin plots show the distribution of EPG per day. Red line: geometric mean. Blue line: arithmetic mean. Green line: median. BL = baseline, 1–28 = days post-treatment.
(TIF)

S2 Fig *A*. *lumbricoides* egg excretion pattern with egg counts derived from Kato-Katz microscopy from participants in the albendazole-ivermectin (A) and albendazole monotherapy (B) treatment group at logarithmic scale. Points represent egg counts from a single sample, lines represent the daily mean eggs per gram (EPG), violin plots show the distribution of EPG per day. Red line: geometric mean. Blue line: arithmetic mean. Green line: median. BL = baseline, 1–28 = days post-treatment.
(TIF)

S3 Fig Sensitivity and specificity analysis per day post-treatment for hookworm results derived from duplicate Kato-Katz slides. Rates are calculated from the ratio of number of positive results vs number of true positives, as defined per the analysis.
(TIF)
