# Supplementary material for: Egg excretion patterns of soil-transmitted helminth infections in humans following albendazole-ivermectin and albendazole treatment
Source: PLoS Negl Trop Dis. 2024 Mar 22;18(3):e0012073. doi: 10.1371/journal.pntd.0012073 (PMC10990175; doi:10.1371/journal.pntd.0012073)
Supplement: S1 Table — (DOCX) [file pntd.0012073.s001.docx]

Supplementary Table 1: Primers and probes used for the detection of *Trichuris trichiura* via real-time quantitative PCR.

| Species, target gene, product size | Primer / Probe | Dye and Quencher | Sequence 5’ to 3’ |
| --- | --- | --- | --- |
| *Trichuris trichiura* |  |  |  |
| Target: 18S, Size: 76bp | TR-F |  | TTGAAACGACTTGCTCATCAACTT |
|  | TR-R |  | CTGATTCTCCGTTAACCGTTGTC |
|  | TR-Pr | FAM-BHQ1 | CGATGGTACGCTACGTGCTTACCATGG |
