# Supplementary figures and images for: Egg excretion patterns of soil-transmitted helminth infections in humans following albendazole-ivermectin and albendazole treatment
Source: PLoS Negl Trop Dis. 2024 Mar 22;18(3):e0012073. doi: 10.1371/journal.pntd.0012073 (PMC10990175; doi:10.1371/journal.pntd.0012073)

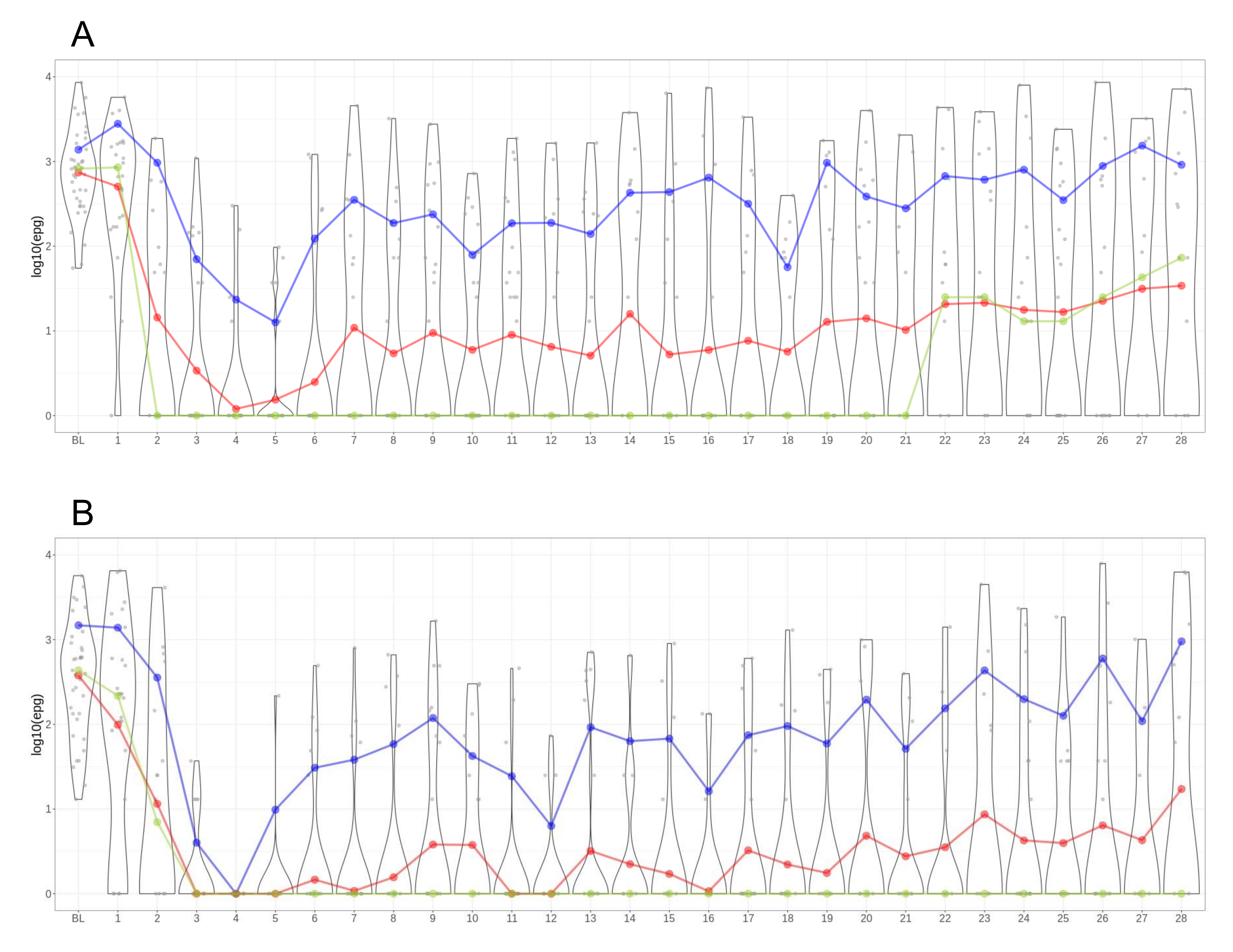

Supplement: S1 Fig — Points represent egg counts from a single sample, lines represent the daily mean eggs per gram (EPG), violin plots show the distribution of EPG per day. Red line: geometric mean. Blue line: arithmetic mean. Green line: median. BL = baseline, 1–28 = days post-treatment. (TIF) [file pntd.0012073.s003.tif]

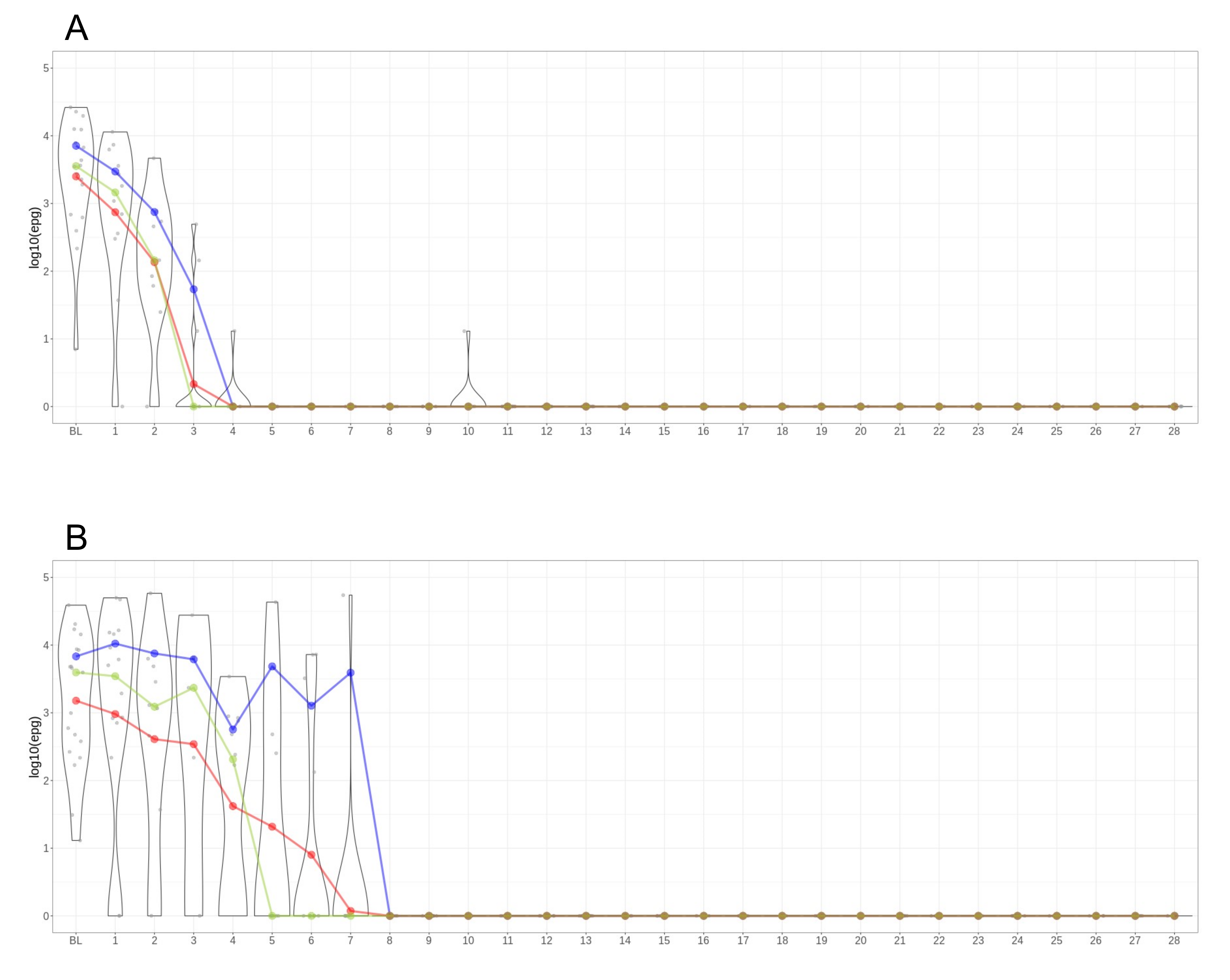

Supplement: S2 Fig — Points represent egg counts from a single sample, lines represent the daily mean eggs per gram (EPG), violin plots show the distribution of EPG per day. Red line: geometric mean. Blue line: arithmetic mean. Green line: median. BL = baseline, 1–28 = days post-treatment. (TIF) [file pntd.0012073.s004.tif]

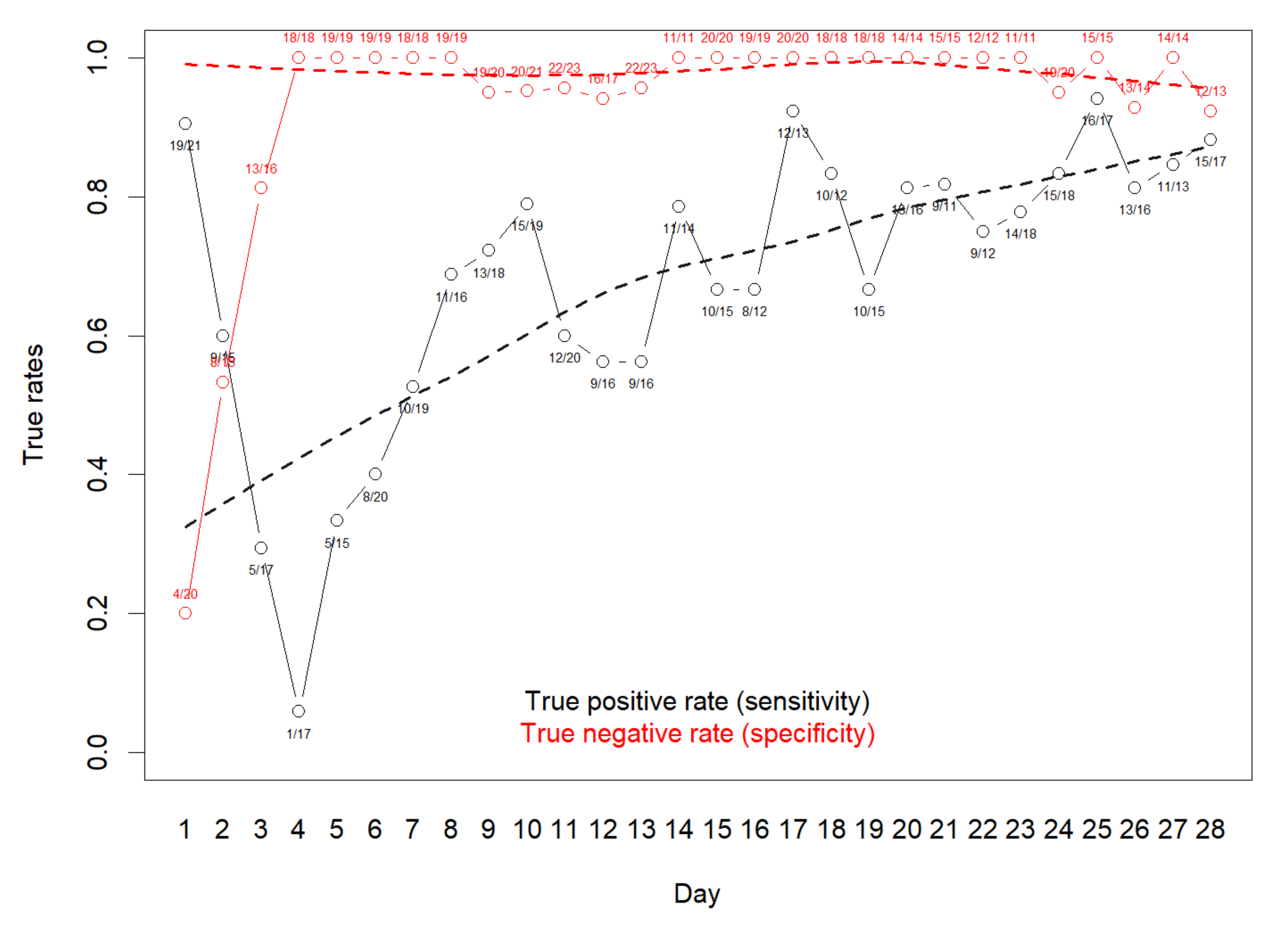

Supplement: S3 Fig — Rates are calculated from the ratio of number of positive results vs number of true positives, as defined per the analysis. (TIF) [file pntd.0012073.s005.tif]
